# The influence of residential distance on time to treatment in ST-elevation myocardial infarction patients

**DOI:** 10.1007/s12471-014-0599-8

**Published:** 2014-10-02

**Authors:** S. Postma, J. H. E. Dambrink, M. J de Boer, A. T. M. Gosselink, J. P. Ottervanger, P. C. Koopmans, J. M. ten Berg, H. Suryapranata, A. W. J. van ’t Hof

**Affiliations:** 1 Diagram, Zwolle, the Netherlands; 2 Department of Cardiology, Isala Hospital, Dokter Van Heesweg 2, 8025AB Zwolle, the Netherlands; 3 UMC St Radboud, Nijmegen, the Netherlands; 4 St Antonius Hospital, Nieuwegein, the Netherlands

**Keywords:** STEMI, Field triage, Referral, Residential distance, Ischaemic time

## Abstract

**Aims:**

To evaluate the relation between residential distance and total ischaemic time in patients with acute ST-elevation myocardial infarction (STEMI).

**Methods:**

STEMI patients were transported to the Isala Hospital Zwolle with the intention to perform primary percutaneous coronary intervention PCI (pPCI) from 2004 until 2010 (*n* = 4149). Of these, 1424 patients (34 %) were referred via a non-PCI ‘spoke' centre (‘spoke’ patients) and 2725 patients (66 %) were referred via field triage in the ambulance (ambulance patients).

**Results:**

A longer residential distance increased median total ischaemic time in ‘spoke’ patients (0–30 km: 228 min, >30-60 km: 235 min, >60-90 km: 264 min, *p* < 0.001), however not in ambulance patients (0–30 km: 179 min, >30-60 km: 175 min, >60-90 km: 186 min, *p* = 0.225). After multivariable linear regression analysis, in ‘spoke’ patients residential distance of >30-60 km compared with 0–30 km was not independently associated with ischaemic time; however, a residential distance of >60-90 km (exp (B) = 1.11, 95 % CI 1.01-1.12) compared with 0–30 km was independently related with ischaemic time. In ambulance patients, residential distance of >30-60 and >60-90 km compared with 0–30 km was not independently associated with ischaemic time.

**Conclusion:**

A longer distance from the patient’s residence to a PCI centre was associated with a small but significant increase in time to treatment in ‘spoke’ patients, however not in ambulance patients. Therefore, referral via field triage in the ambulance did not lead to a significant increase in time to treatment, especially at long distances (up to 90 km).

## Introduction

Strategies to reduce time delays in ST-segment elevated myocardial infarction (STEMI) patients are currently of great interest, since shorter time delays improve outcome [1–5]. Transportation delay is a time delay which is mainly dependent on the type of transport, mode of referral, geographical area, weather conditions, traffic and distance. In urban areas, ambulance transport is the transport of choice and in rural areas air transport usually facilitates the transfer of STEMI patients. Previous studies have shown that optimising logistics by field triage in the ambulance can help to reduce time to treatment and improve outcomes compared to referral via a non-percutaneous coronary intervention (PCI) spoke centre (peripheral centre) [5–12]. Only a few published data exist on the effect of geographical area [8, 13–16], weather conditions [16], traffic [16] and distance [5, 16–19] on time to treatment.

In the Netherlands, distance to a PCI centre (hub centre: specialised central interventional centre) was one of the reasons to expand primary PCI to more hospitals, including those without on-site cardiac surgery. It was expected that with more PCI centres transportation delay might decrease and clinical outcomes might improve. However, recently Concannon et al. demonstrated that introducing new PCI centres did not help patients gain access to timely PCI [20]. Therefore, we have investigated the relation between residential distance and total ischaemic time in STEMI patients referred to a large tertiary PCI centre. In addition, the effect of residential distance on total ischaemic time was assessed in patients who were referred via a non-PCI spoke (spoke) centre and in patients referred via field triage in de ambulance.

## Methods

### Population

Since the early 1990s, STEMI patients referred to the Isala Hospital Zwolle were treated by primary PCI (pPCI). To improve the logistics of STEMI patients, the field triage project was initiated. This has gradually been implemented in the region, starting in 1998 until all ambulances were part of the field triage project. During the project all consecutive STEMI patients who were transported to the PCI centre with the intention to perform pPCI, from 2004 until 2010, were prospectively registered in a dedicated database. Criteria for the diagnosis of STEMI were: 1) history of cardiac symptoms of at least 30 min in the last 12 h or between 12 and 24 h if they had persistent symptoms with evidence of ongoing ischaemia; 2) elevated levels of creatine kinase (CK) or creatine kinase-MB (CK-MB) and 3) concurrent electrocardiogram (ECG) changes: ST-segment elevation of >0.1 mV in at least two adjacent electrocardiogram leads [21].

The residential distance to the nearest PCI centre via the motorway was computerised using the postal codes of the patient’s residence and the PCI centre. Subsequently three groups were formed according to distance: 1) 0–30 km, 2) >30-60 km and 3) >60-90 km. Furthermore, a subdivision by type of triage was performed. Patients were transferred via a referral spoke centre in the network (spoke group) or via field triage in the ambulance (ambulance group).

Patients were excluded if the distance from the patient’s residence to the PCI centre could not be assessed or was >90 km (outer boundary of referring area).

### Triage for pPCI

We hypothesised that the effect of distance on outcome might be different for spoke patients versus ambulance patients, as referral logistics are different between the two groups. Spoke patients are transported twice: the first time to bring the patient from their residence to the nearest spoke centre and a second time to bring the patient to the PCI centre after diagnosis of myocardial infarction in the spoke centre. Conversely, ambulance patients are transported only once via the shortest and fastest possible way and immediately after myocardial infarction diagnosis at the patient’s residence.

Spoke group: If the ambulance was not equipped with field triage equipment, the ambulance went to the nearest spoke centre where diagnosis and triage was performed. If the ECG performed upon arrival was diagnostic for STEMI, patients were transported to the catheterisation laboratory of the PCI centre as soon as possible, preferably by using the same ambulance.

Ambulance group: The algorithm of field triage has been described previously [17]. In brief, after patients had dialled the emergency number, they were triaged in the ambulance. An ECG was performed by highly trained paramedics followed by interpretation by the computerised algorithm. If a diagnosis of STEMI was made, the ambulance went straight to the catheterisation laboratory of the PCI centre, bypassing the emergency departments of nearby spoke centres.

Walk-ins at the PCI centre were excluded since they did not receive field triage.

### pPCI procedure

In both situations, the staff of the catheterisation laboratory of the PCI centre was pre-informed about the estimated time of arrival of the patient and was activated well before the arrival of the patient. If the staff lived more than 30 min away from the PCI centre, they had to stay in the PCI centre when on call. All patients were treated pre-hospital with an intravenous bolus of 5000 IU of unfractionated heparin and 500 mg aspirin intravenously. During the study period the administration of clopidogrel on top of aspirin and heparin as pre-hospital treatment was implemented at 1 July 2006. The administration of GP IIb/IIIa blockers in the pre-hospital phase was left at the discretion of the referring physicians.

### Time intervals

Four different time intervals were evaluated: 1) Time from symptom onset to infarct diagnosis (time of diagnostic ECG) either in the ambulance or at a spoke centre (symptom onset to diagnosis); 2) Time from diagnosis till arrival at the PCI centre (diagnosis to door PCI); 3) Time from arrival at the PCI centre to balloon inflation (door to balloon) and 4) Total ischaemic time defined as the time from symptom onset to balloon inflation.

Patients were excluded if the total ischaemic time could not be assessed.

### Statistical analysis

Statistical analysis was performed with SPSS 20.0. Continuous data were expressed as mean ± SD or median and interquartile range. Categorical data were presented as percentages. A Kruskal-Wallis test was used for continuous data, since they were non-Gaussian distributed. A Pearson’s Chi-square test was used for categorical data. The relationship between total ischaemic time and residential distance for each patient was assessed using Spearman’s correlation. Linear regression analysis was performed to estimate the effect of residential distance on total ischaemic time. To assess whether the mode of referral might interfere with the relationship between residential distance and total ischaemic time, interaction testing was performed. For the regression analysis and the interaction testing total ischaemic time was log transformed, since this time interval was non-Gaussian distributed.

All above-described statistical tests were two-sided. In all statistical analyses p values <0.05 were considered to be statistically significant.

The study was conducted according to the principles of the Declaration of Helsinki and the protocol was approved by the local institutional review board. No extramural funding was used to support this work. The authors are solely responsible for the design and conduct of this study, all study analyses and drafting and editing of the paper.

## Results

From 2004 until 2010, 5285 patients were referred to our hospital with the intention to perform pPCI. A total of 349 patients (6.6 %) were walk-ins at the PCI centre, in 184 patients the residential distance could not be assessed (3.5 %) and 603 patients (11.4 %) were excluded because the total ischaemic time could not be assessed.

**Fig. 1:**
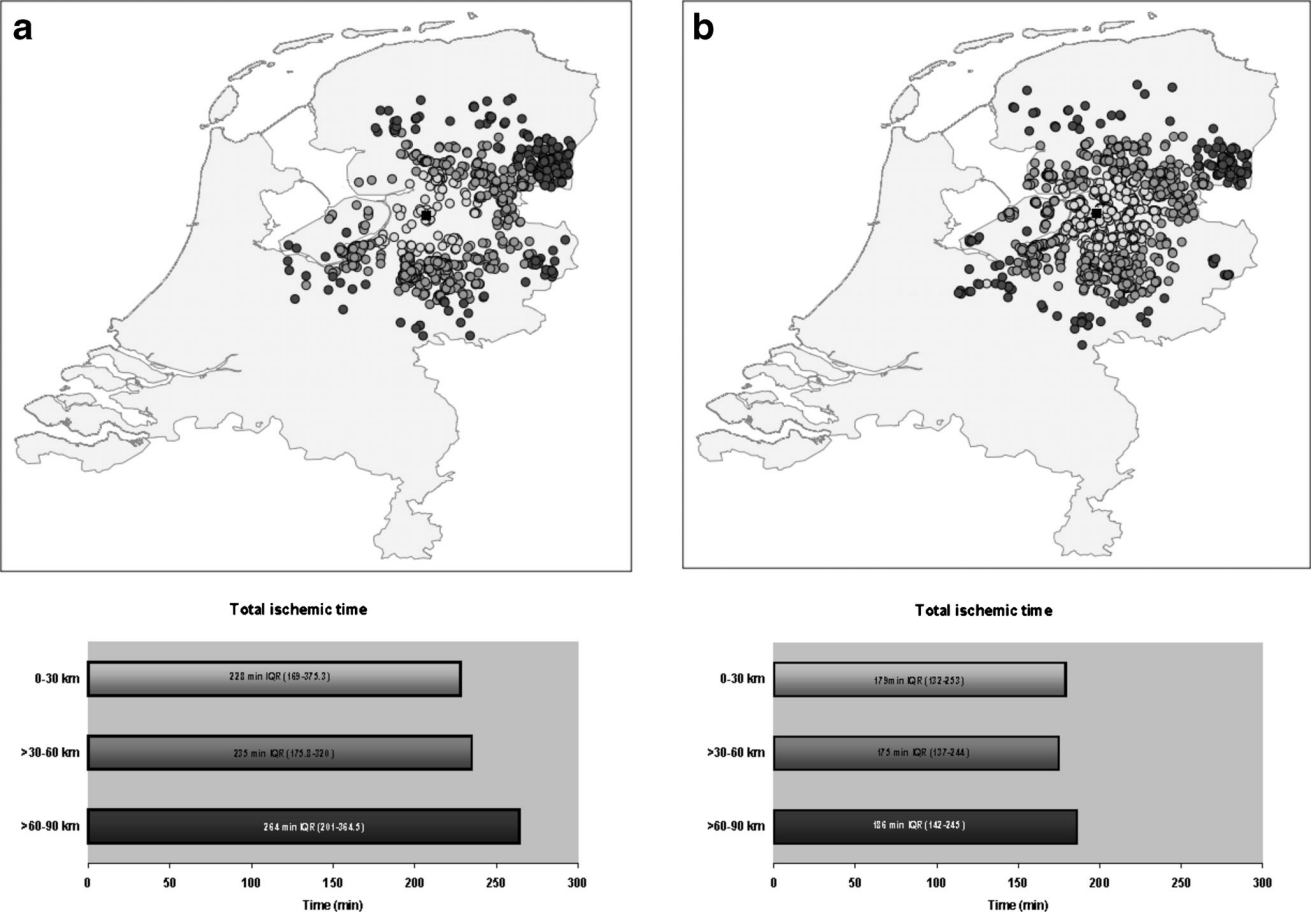
Distribution of residential distance and total ischaemic time Above the distance from the patient’s residence to the PCI centre is illustrated on a map of the Netherlands for the spoke group (**a**) and the ambulance group (**b**). Black dot: Isala Hospital Zwolle, light grey dots: patients living 0–30 km from the PCI centre, grey dots: patients living >30-60 km from the PCI centre, dark grey dots: patients living >60-9 0 km from the PCI centre. Below the total ischaemic time is shown for the spoke group (**a**) and the ambulance group (**b**) for patients living 0–30 km, >30-60 km and >60-90 km from a PCI centre

Of the remaining 4149 patients, 1424 patients (34 %) were referred via a spoke centre and 2725 (66 %) via field triage in the ambulance. Fig. 1a and b illustrate the distance from the patient’s residence to the PCI centre for the spoke group and the ambulance group on a map of the Netherlands. Spoke patients mainly lived between 30–90 km away from the PCI centre, while ambulance patients mostly lived within a residential distance of 0–60 km.

There was a significant interaction effect between residential distance and type of triage on total ischaemic time (*p* = 0.038). Therefore, the results are presented separately for the two groups.

### Spoke group

The baseline characteristics of spoke patients are described in Table 1. The presence of Killip class >1 and incidence of previous CABG decreased with distance from patient’s residence to the PCI centre. The correlation between residential distance and total ischaemic time was weak although significant (r = 0.078, *p* = 0.003). Furthermore, median total ischaemic time increased with distance as well as the other described time intervals (Table 2 and Fig. 1a). After multivariable linear regression analysis residential distance of >30-60 km (exp (B) = 0.98, 95 % CI 0.90-1.06) compared with 0–30 km was not independently associated with ischaemic time, however a residential distance of >60-90 km (exp (B) = 1.11, 95 % CI 1.01-1.12) compared with 0–30 km was independently related with ischaemic time.

**Table 1:** Baseline characteristics

	Spoke group				Ambulance group			
	Distance	Distance	Distance	*P*-value	Distance	Distance	Distance	*P*-value
	0–30 km	>30–60 km	>60–90 km		0–30 km	>30–60 km	>60–90 km	
Characteristic	*n* = 134	*n* = 978	*n* = 312		*n* = 1246	*n* = 1263	*n* = 216	
Age years ± SD	64.41 ± 12.94 (*n* = 134)	63.48 ± 12.81 (*n* = 978)	62.59 ± 11.76 (*n* = 312)	0.350	63.52 ± 13.00 (*n* = 1246)	63.40 ± 12.28 (*n* = 1263)	63.02 ± 12.09 (*n* = 216)	0.813
Male gender	98/134 (73.1 %)	674/978 (68.9 %)	226/312 (72.4 %)	0.358	921/1246 (73.9 %)	943/1263 (74.7 %)	159/216 (73.6 %)	0.891
Previous MI	16/131 (12.2 %)	111/975 (11.4 %)	36/312 (11.5 %)	0.961	110/1243 (8.8 %)	118/1254 (9.4 %)	12/215 (5.6 %)	0.189
Previous CABG	7/131 (5.3 %)	32/978 (3.3 %)	3/312 (1.0 %)	0.026	37/1245 (3.0 %)	36/1259 (2.9 %)	7/215 (3.3 %)	0.947
Previous PCI	13/131 (9.9 %)	93/976 (9.5 %)	33/310 (10.6 %)	0.846	125/1245 (10.0 %)	97/1256 (7.7 %)	15/215 (7.0 %)	0.078
Previous CVA	5/131 (3.8 %)	29/978 (3.0 %)	9/312 (2.9 %)	0.855	39/1245 (3.1 %)	37/1255 (2.9 %)	7/215 (3.3 %)	0.950
Hypertension	44/131 (33.6 %)	362/974 (37.2 %)	111/311 (35.7 %)	0.686	437/1241 (35.2 %)	420/1251 (33.6 %)	63/215 (29.3 %)	0.220
Diabetes mellitus	14/131 (10.7 %)	118/975 (12.1 %)	42/312 (13.5 %)	0.690	131/1245 (10.5 %)	128/1258 (10.2 %)	18/216 (8.3 %)	0.617
Hypercholesterolaemia	31/129 (24.0 %)	209/954 (21.9 %)	77/305 (25.2 %)	0.455	254/1208 (21.0 %)	245/1220 (20.1 %)	42/209 (20.1 %)	0.837
Smoking	57/130 (43.8 %)	381/955 (39.9 %)	144/305 (47.2 %)	0.070	512/1227 (41.7 %)	541/1243 (43.5 %)	83/212 (39.2 %)	0.410
Anterior MI	56/132 (42.4 %)	409/970 (42.2 %)	139/307 (45.3 %)	0.627	489/1205 (40.6 %)	530/1229 (43.1 %)	95/212 (44.8 %)	0.315
Killip class >1	12/133 (9.0 %)	91/973 (9.4 %)	11/311 (3.5 %)	0.004	96/1243 (7.7 %)	83/1260 (6.6 %)	15/216 (6.9 %)	0.540
TIMI risk score (mean ± SD, median (IQR))	3.09 ± 2.29	3.16 ± 2.27	2.95 ± 2.09	0.592	2.79 ± 2.16	2.78 ± 2.09	2.81 ± 2.46	0.806
	3.0 (1.0 - 5.0)	3.0 (1.0 - 5.0)	3.0 (1.0 - 4.0)		3.0 (1.0 - 4.0)	3.0 (1.0 - 4.0)	2.0 (1.0 - 4.0)	
	(n = 119)	(n = 839)	(n = 214)		(n = 1108)	(n = 1144)	(n = 1144)	
GPIIb/IIIa blocker*a*	43/134 (32.1 %)	278/978 (28.4 %)	73/312 (23.4 %)	0.109	218/1246 (17.5 %)	219/1263 (17.3 %)	26/216 (12.0 %)	0.129

^*a*^ *administered in the acute phase*

CABG: coronary artery bypass graft, CVA: cerebrovascular accident, IQR: interquartile range, GPIIb/IIIa: Glycoprotein IIb/IIIa; MI myocardial infarction, PCI: percutaneous coronary intervention, SD: standard deviation, TIMI: thrombolysis in myocardial infarction

**Table 2:** Time intervals

	Spoke group				Ambulance group			
	Distance 0–30 km	Distance >30–60 km	Distance >60–90 km	*P*-value	Distance 0–30 km	Distance >30–60 km	Distance >60–90 km	*P*-value
Time intervals	*n* = 134	*n* = 978	*n* = 312		*n* = 1246	*n* = 1263	*n* = 216	
Ischaemic time (median, IQR)	228 (169–375.3)	235 (175.8–320)	264 (201–364.5)	<0.001	179 (132–253)	175 (137–244) )	186 (142–245)	0.225
	(*n*=134)	(*n*=978)	(*n*=312)		(*n*=1246)	(*n*=1263	(*n*=216)	
SO-diagnosis (median, IQR)	86 (48–217)	112 (62–196)	137.5 (72.3–223)	0.039	77 (44–142)	74.5 (42–133)	71 (39–135)	0.211
	(*n*=119)	(*n*=859)	(*n*=268)		(*n*=1154)	*n*=1186)	(*n*=205)	
Diagnosis-door PCI (median, IQR)	73.5 (523–109.5)	74 (56–100)	93 (74.5–118)	<0.001	41 (30–53)	53 (42–65.5)	66 (50–82.3)	<0.001
	(*n*=120)	(*n*=861)	(*n*=273)		(*n*=1107)	(*n*=1169)	(*n*=205)	
D2B (median, IQR)	40 (25–69)	41 (27–60)	35 (25–53)	0.034	50 (35–75)	42 (29–60)	40 (29–60.3)	<0.001
	(*n*=114)	(*n*=844)	(*n*=290)		(*n*=1071)	(*n*=1125)	(*n*=190)	

D2B: door to balloon, IQR: interquartile range, PCI: percutaneous coronary intervention, SO: symptom onset

### Ambulance group

The baseline characteristics of ambulance patients are described in Table 1. The correlation between residential distance and total ischaemic time was not significant (r = 0.017, *p* = 0.382). The time from diagnosis to door PCI increased (*p* < 0.001) and the door to balloon time decreased (*p* < 0.001) (Table 2 and Fig. 1b). After multivariable linear regression analysis a residential distance of >30-60 km (exp (B) = 1.00, 95 % CI 0.97-1.04) and of >60-90 km (exp (B) = 1.06, 95 % CI 0.99-1.13) compared with 0–30 km were not independently related to ischaemic time.

## Discussion

Our study is the largest of its kind demonstrating that living further away from a PCI centre increases time to treatment in patients referred via a spoke centre, but to a lesser extent in patients who were immediately transported after field triage in the ambulance. Overall the increase in median total ischaemic time with longer residential distance was modest (30–90 km: 36 min (spoke) vs. 7 min (ambulance)). This limited effect of residential distance on total ischaemic time can partly be explained by the reduced door to balloon time with longer residential distance. A longer transportation delay gives the opportunity to prepare the cath-lab for PCI, after a call from the ambulance or spoke centre. These findings imply that a substantial increase in residential distance can be covered with only a modest increase in transportation time and suggests that prolonged transportation distances have a limited effect on outcome, when other components of the total ischaemic time are optimally organised. This was clearly found when patients were diagnosed and immediately transported after pre-hospital triage in the ambulance: for 30–60 km residential distance from the PCI centre transportation took 74 min (median) for a spoke patient as compared with 53 min (median) for an ambulance patient (Table 2). In this regard it should be emphasised that residential distance as mentioned in the study is calculated as the shortest distance by motorway from the patient’s residence to the PCI centre. Ambulance-triaged patients were transported via this shortest distance whereas spoke patients travelled longer distances. Firstly, on the way to the spoke centre and secondly, on the way from the spoke centre to the PCI centre, consequently resulting in a longer time to diagnosis and a long inter-hospital transportation time.

Until now, only few data are available on the relation between residential distance and total ischaemic time. Recently we showed that field triage in the ambulance may reduce the negative effect of living at a longer distance from a PCI centre [5]. Beri et al. have demonstrated that longer distances did not result in any significant transfer delay [22]. However, they did not investigate the effect of short versus long distance, but the effect of pPCI versus fibrinolytic therapy at long distance.

Besides referring patients directly after field triage in the ambulance to a PCI centre, expansion of primary PCIs to more hospitals is also a widely discussed option to improve treatment delay [20, 23].

We believe expansion of PCI centres may play less of a role in the overall improvement of timely treatment of STEMI patients, since our results demonstrate that residential distance is only weakly associated with total ischaemic time if patients are transferred via field triage in the ambulance. Furthermore, the number, availability and expertise of the interventional cardiologists plays also an important role in providing timely access to PCI as well as the expertise of ambulance personnel. In addition, in the future more attention is needed for changes in PCI capacity and the effects of these changes on outcome measures as well as on the selection of high-risk patients for transfer.

### Limitations

Firstly, the postal code of the patients’ residence was used as a proxy for the place where the ambulance picked up the patient. Information on the exact distance between the patient’s residence and the place where the ambulance picked up the patient was not available. However, a random sample of 716 cases revealed that >80 % of the patients were picked up <5 km from their residence. Secondly, since the project was not randomised and was spread over more than 10 years, the risk of unknown confounders exists. Thirdly and perhaps the most important limitation is the fact that our study was performed in a small country with a flat landscape where ambulance triage for STEMI patients is optimised, where distances between the patient’s residence and PCI centres are surmountable to treat most patients according to the ACC/AHA and ESC guidelines and where traffic and weather conditions are no major issue. More research is needed to investigate whether comparable results can be achieved in other areas of the world.

## Conclusions

A longer distance from the patient’s residence to a PCI centre was associated with a small but significant increase in time to treatment in patients referred via a non-PCI spoke centre, although this association was weak. In patients who were referred via field triage in the ambulance there was no significant association between residential distance and time to treatment. Therefore, referral via field triage in the ambulance did not lead to a significant increase in time to treatment, especially at long distances (up to 90 km).
